# Riboflavin Accumulation and Molecular Characterization of cDNAs Encoding Bifunctional GTP Cyclohydrolase II/3,4-Dihydroxy-2-Butanone 4-Phosphate Synthase, Lumazine Synthase, and Riboflavin Synthase in Different Organs of *Lycium chinense* Plant

**DOI:** 10.3390/molecules191117141

**Published:** 2014-10-24

**Authors:** Pham Anh Tuan, Shicheng Zhao, Jae Kwang Kim, Yeon Bok Kim, Jingli Yang, Cheng Hao Li, Sun-Ju Kim, Mariadhas Valan Arasu, Naif Abdullah Al-Dhabi, Sang Un Park

**Affiliations:** 1 Department of Crop Science, Chungnam National University, 99 Daehak-Ro, Yuseong-gu, Daejeon 305-764, Korea; E-Mails: tuan_pham_6885@yahoo.com (P.A.T.); zhaoshicheng@msn.com (S.Z.); yeonbokkim@hanmail.net (Y.B.K.); 2 Division of Life Sciences, College of Life Sciences and Bioengineering, Incheon National University, Incheon 406-772, Korea; E-Mail: kjkpj@incheon.ac.kr; 3 State Key Laboratory of Tree Genetics and Breeding, Northeast Forestry University, Harbin 150040, China; E-Mails: yifan85831647@sina.com (J.Y.); chli0@163.com (C.H.L.); 4 Department of Bio-Environmental Chemistry, Chungnam National University, 99 Daehak-ro, Yuseong-gu, Daejeon 305-764, Korea; E-Mail: kimsunju@cnu.ac.kr; 5 Department of Botany and Microbiology, Addiriyah Chair for Environmental Studies, College of Science, King Saud University, P.O. Box 2455, Riyadh 11451, Saudi Arabia; E-Mail: mvalanarasu@gmail.com; 6 Visiting Professor Program (VPP), King Saud University, P.O. Box 2455, Riyadh 11451, Saudi Arabia

**Keywords:** GTP cyclohydrolase II/3,4-dihydroxy-2-butanone-4-phosphate synthase, lumazine synthase, riboflavin synthase, *Lycium chinense*, riboflavin

## Abstract

Riboflavin (vitamin B2) is the precursor of flavin mononucleotide and flavin adenine dinucleotide—essential cofactors for a wide variety of enzymes involving in numerous metabolic processes. In this study, a partial-length cDNA encoding bifunctional GTP cyclohydrolase II/3,4-dihydroxy-2-butanone-4-phosphate synthase (LcRIBA), 2 full-length cDNAs encoding lumazine synthase (LcLS1 and LcLS2), and a full-length cDNA encoding riboflavin synthase (LcRS) were isolated from *Lycium chinense*, an important traditional medicinal plant. Sequence analyses showed that these genes exhibited high identities with their orthologous genes as well as having the same common features related to plant riboflavin biosynthetic genes. LcRIBA, like other plant RIBAs, contained a DHBPS region in its N terminus and a GCHII region in its C-terminal part. LcLSs and LcRS carried an N-terminal extension found in plant riboflavin biosynthetic genes unlike the orthologous microbial genes. Quantitative real-time polymerase chain reaction analysis showed that 4 riboflavin biosynthetic genes were constitutively expressed in all organs examined of *L. chinense* plants with the highest expression levels found in the leaves or red fruits. LcRIBA, which catalyzes 2 initial reactions in riboflavin biosynthetic pathway, was the highest transcript in the leaves, and hence, the richest content of riboflavin was detected in this organ. Our study might provide the basis for investigating the contribution of riboflavin in diverse biological activities of *L. chinense* and may facilitate the metabolic engineering of vitamin B2 in crop plants.

## 1. Introduction

**Scheme 1:**
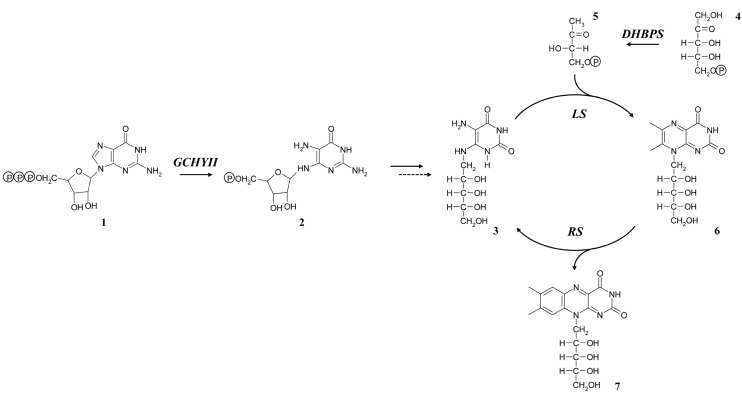
The proposed riboflavin biosynthetic pathway in plants.

Riboflavin is an indispensable vitamin (vitamin B2) for humans and has been reported to play roles in protecting against cataract, cancer, and cardiovascular diseases [1,2,3]. It is the precursor of the coenzymes flavin mononucleotide and flavin adenine dinucleotide, and is also involved in numerous physiological processes involving light sensing, bioluminescence, and DNA repair [4,5,6]. In all organisms, the biosynthesis of 1 riboflavin molecule requires 1 molecule of GTP and 2 molecules of ribulose 5-phosphate (Scheme 1) [7,8]. The imidazole ring of GTP is hydrolytically cleaved by GTP cyclohydrolase II (GCHYII) to form 2,5-diamino-6-ribosylamino-4(3*H*)-pyrimidinone-5'-phosphate, which is then converted to 5-amino-6-ribitylamino-2,4(1*H*,3*H*)-pyrimidinedione by several reactions involving deamination, side chain reduction, and dephosphorylation [9]. Subsequently, lumazine synthase (LS) catalyzes the condensation of 5-amino-6-ribitylamino-2,4(1*H*,3*H*)-pyrimidinedione with 3,4-dihydroxy-2-butanone-4-phosphate, which is obtained from ribulose 5-phosphate by 3,4-dihydroxy-2-butanone-4-phosphate synthase (DHBPS), yielding 6,7-dimethyl-8-ribityllumazine [10]. The final step of the riboflavin biosynthetic pathway is mediated by riboflavin synthase (RS), which catalyzes the disproportionation of 6,7-dimethyl-8-ribityllumazine, affording riboflavin and 5-amino-6-ribitylamino-2,4(1*H*,3*H*)-pyrimidinedione, which is recycled in the biosynthetic pathway [11]. Over the past 15 years, several researchers have investigated riboflavin biosynthesis by cloning a bifunctional protein with GCHYII and DHBP in *Arabidopsis* [9]; characterization of LS and RS has been conducted in spinach, tobacco, and bitter melon [12,13]. Recently, the availability of next-generation sequencing technologies has allowed the extensive investigation of the genes related to the riboflavin biosynthetic pathway in plants.

1: GTP; 2: 2,5-diamino-6-ribosylamino-4(3*H*)-pyrimidinone-5'-phosphate; 3: 5-amino-6-ribitylamino-2,4 (1*H*,3*H*)-pyrimidinedione; 4: ribulose-5-phosphate; 5: 3,4-dihydroxy-2-butanone-4-phosphate; 6: 6,7-dimethyl-8-ribityllumazine; 7: riboflavin; GCHYII: GTP cyclohydrolase II; DHBPS: 3,4-dihydroxy-2-butanone-4-phosphate synthase; LS: lumazine synthase; RS: riboflavin synthase.

*Lycium chinense*, belonging to the family Solanaceae, has been used for more than 2000 years in traditional Chinese medicine as a medical herb for anti-aging purposes and as a nourishing ingredient to reduce the risk of arteriosclerosis and arterial hypertension [14,15]. *L. chinense* contains a wide range of functional components that have beneficial effects on health, such as betain, ascorbic acid, flavonoids, carotenoids, and alkaloids [16,17,18,19]. Therefore, it is known to possess many biological activities, including antioxidant, antiallergic, and anti-inflammatory activities [17,20,21]. In addition, *L. chinense* fruits were reported to reduce myofibroblast-like cell proliferation and to induce hepatic fibrosis [19,22].

Information on genes related to the riboflavin biosynthetic pathway in planta was obtained by isolating a partial-length cDNA encoding bifunctional GCHYII/DHBP (LcRIBA), two full-length cDNAs encoding LS (LcLS1 and LcLS2), and a full-length cDNA encoding RS (LcRS) from *L. chinense*. In addition, the transcript levels of four riboflavin biosynthetic genes and accumulation of riboflavin were investigated in the roots, stems, leaves, flowers, green fruits, and red fruits of *L. chinense*.

## 2. Results and Discussion

### 2.1. Sequence Analyses of Riboflavin Biosynthetic Genes from L. chinense

**Figure 1:**
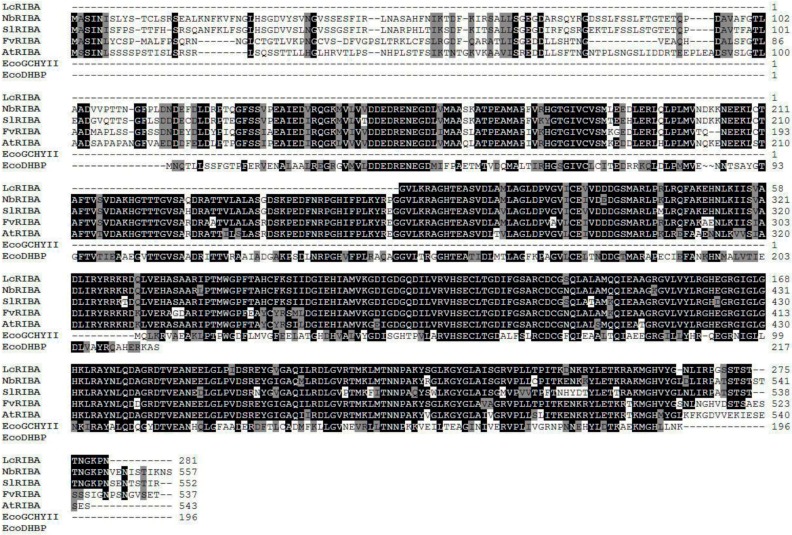
Multiple alignments of the amino acid sequences of LcRIBA with other RIBAs.

In *Escherichia coli*, the two initial reactions in riboflavin biosynthesis are catalyzed by monofunctional GCHII and DHBPS proteins [23,24]. Interestingly, a bifunctional RibA gene that has both GCHII and DHBPS activities was found in *Arabidopsis* [9]. In this study, a bifunctional GCHII/DHBPS was also found in *L. chinense* (LcRIBA), which consisted of 846 bps encoding a partial open reading frame (ORF) of 281 amino acids. A BLAST search at the amino acid level showed that LcRIBA shared high homology with other plant RIBAs. Specifically, LcRIBA shared 93% identity and 97% similarity with *Nicotiana benthamiana* RIBA, 90% identity and 93% similarity with *Solanum lycopersicum* RIBA, 90% identity and 96% similarity with *Fragaria vesca* RIBA, and 88% identity and 96% similarity with *Arabidopsis thaliana* RIBA. The alignment of plant RIBAs and *E. coli* monofunctional GCHII and DHBPS revealed that (Figure 1) plant RIBAs encode a bifunctional protein containing DHBPS and GCHII regions in their N- and C-terminal parts, respectively.

**Figure 2:**
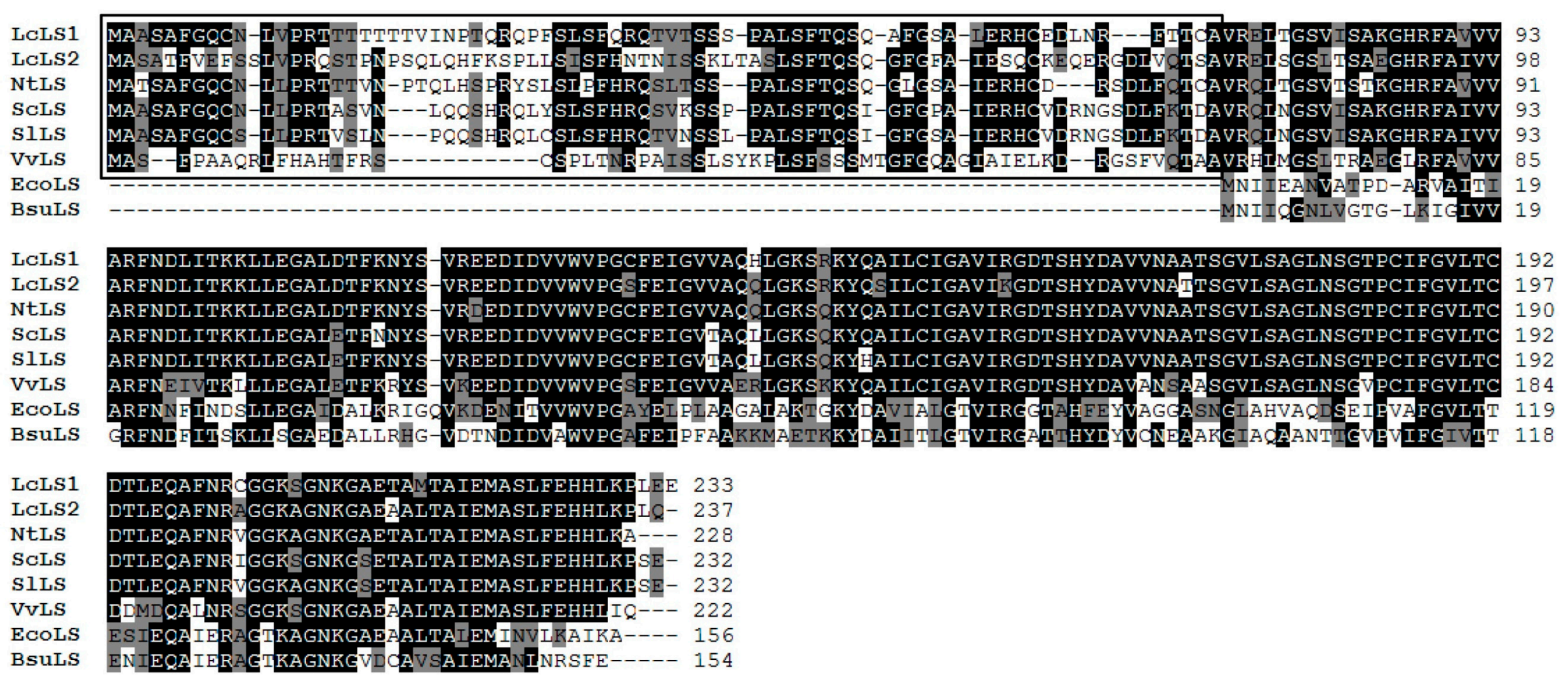
Multiple alignments of the amino acid sequences of LcLS1 and LcLS2 with other LSs.

LcLS1 consisted of 702 bp encoding a protein of 233 amino acids (predicted molecular mass (MM), 25.07 kDa) and LcLS2 consisted of 714 bp encoding a protein of 237 amino acids (predicted MM, 25.41 kDa). LcLS1 and LcLS2 shared 83% and 76% identity with *Nicotiana tabacum* LS; 82% and 73% with *Solanum chacoense* LS; 82%and 74% with *S. lycopersicum* LS; and 68% and 74% with *Vitis vinifera* LS, respectively. Similar to other plant LSs, LcLS1 and LcLS2 also carried N-terminal extensions having a length of 73 amino acids and 78 amino acids, respectively, compared to the microbial LSs from *E. coli* and* Bacillus subtilis* (Black boxes; Figure 2) [12,25].

Identical residues are indicated by a black background, and similar residues are shaded with a gray background. NtRIBA, *Nicotiana benthamiana* RIBA (AB538870); SlRIBA, *Solanum lycopersicum* RIBA (NM_001247847); FvRIBA, *Fragaria vesca* RIBA (XM_004288112); AtRIBA, *Arabidopsis thaliana* RIBA (AJ000053); EcoGCHYII, *Escherichia coli* GCHYII (CCJ43810), EcoDHBP, *Escherichia coli* DHBP (CCJ45664).

The black box shows the N-terminal extension found in plant riboflavin biosynthetic genes compared with that found in the homologous microbial enzymes. Identical residues are indicated by a black background, and similar residues are shaded with a gray background. NtLS, *Nicotiana tabacum* LS (AF422802); ScLS, *Solanum chacoense* LS (EU526014); SlLS, *Solanum lycopersicum* LS (XM_004252996); VvLS, *Vitis vinifera* LS (XM_002280391); EcoLS, *Escherichia coli* LS (YP_001729321); BsuLS, *Bacillus subtilis* LS (WP_003223915).

**Figure 3:**
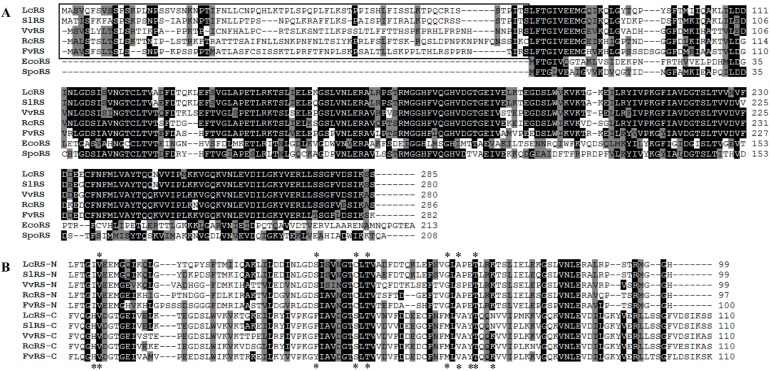
Multiple alignments of amino acid sequences (**A**) or the N- and C-terminal domains (**B**) of LcRS with other RSs.

The black box shows the N-terminal extension found in the plant riboflavin biosynthetic genes compared with that found in the homologous microbial enzymes. Residues proposed to interact with the substrate of RS are marked by asterisks. Identical residues are indicated by a black background, and similar residues are shaded with a gray background. N, N-terminal domain; C, C-terminal domain. SlRS, *Solanum lycopersicum* RS (XM_004243294); VvRS, *Vitis vinifera* RS (XM_002273993); RcRS, *Ricinus communis* RS (XM_002517874); FvRS, *Fragaria vesca* RS (XM_004301399); EcoRS, *Escherichia coli* RS (CAA48861); SpoRS, *Schizosaccharomyces pombe* RS (AAM28201).

LcRS was 858-bp long encoding a protein of 285 amino acids with a predicted MM of 31.32 kDa. LcRS shared 84% identity and 89% similarity with *S. lycopersicum* RS, 66% identity and 74% similarity with *V. vinifera* RS, 63% identity and 73% similarity with *Ricinus communis* RS, and 68% identity and 78% similarity with *F. vesca* RS. LcRS contained a 76-amino-acid N-terminal extension—found in plant riboflavin biosynthetic genes—unlike in *E. coli* RS and *Schizosaccharomyces pombe* RS (Black box; Figure 3A).

Moreover, there was a close similarity between N- and C-terminal domains of RS genes (Figure 3B). Each domain was thought to bind one substrate molecule in a shallow cavity [12]. Residues proposed to interact with the substrate of RS are marked by asterisks in Figure 3B [26].

### 2.2. Expression Levels of LcRIBA, LcLS1, LcLS2, and LcRS in Different Organs of L. chinense

**Figure 4:**
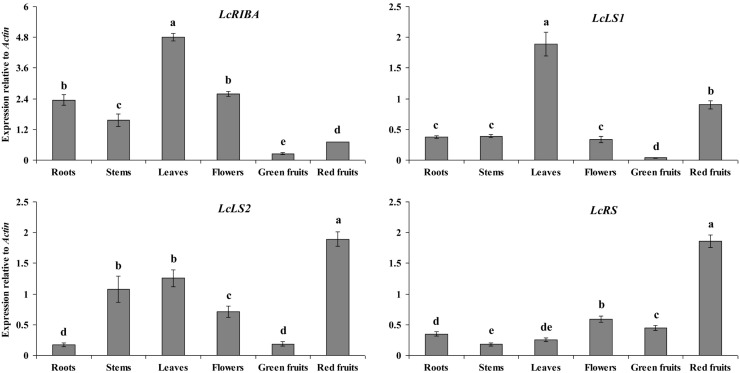
Expression levels of* LcRIBA*,* LcLS1*,* LcLS2*, and *LcRS* in different organs of *L. chinense*.

Quantitative real-time PCR (qRT-PCR) analysis results of the expression patterns of *LcRIBA, LcLS1, LcLS2,* and* LcRS* in the roots, stems, leaves, flowers, green fruits, and red fruits of *L. chinense* are shown in Figure 4. These genes were constitutively expressed in all organs examined with different expression patterns. Transcription of *LcRIBA* was the highest in the leaves; moderate in the roots, stem, and flowers; and weak in the green and red fruits. *LcLS1* and* LcLS2* were differentially expressed in *L. chinense* plants. The highest mRNA level of *LcLS1* was found in the leaves, followed by that in the red fruits, while only low levels were detected in the roots, stems, flowers, and green fruits. *LcLS2* exhibited the highest transcription level in the red fruits, moderate levels in the stems and leaves, and only low levels in the roots and red fruits. A substantially higher level of *LcRS* mRNA was detected in the red fruits than in the roots, stems, leaves, flowers, and green fruits.

The height of each bar and the error bars show the mean and standard errors, respectively, from three independent measurements. The letters a, b, c, d, and e indicate significant differences at the 5% level by Duncan’s multiple range test.

### 2.3. Anaysis of Riboflavin Content in Different Organs of L. chinense

**Figure 5:**
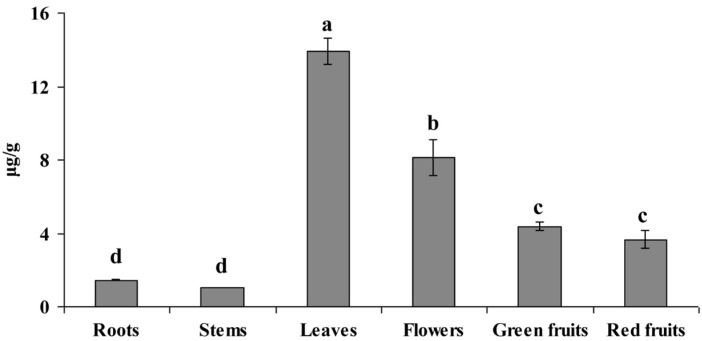
Riboflavin content in different organs of *L. chinense*.

The same plant materials as those used for qRT-PCR were used for HPLC analysis of riboflavin in *L. chinense* (Figure 5). The richest content of riboflavin (13.91 μg/g dry weight) was found in the leaves. The flower also contained a high content of riboflavin (8.14 μg/g), while only small amounts of riboflavin were found in the roots (1.45 μg/g) and stem (1.03 μg/g). Statistical analysis suggested that there was no significant difference in riboflavin accumulation between green and red fruits (4.36 μg/g and 3.66 μg/g, respectively).

The height of each bar and the error bars show the mean and standard errors, respectively, from three independent measurements. The letters a, b, c, and d indicate significant differences at the 5% level by Duncan’s multiple range test.

In this study, four genes related to the riboflavin biosynthetic pathway, *LcRIBA, LcLS1, LcLS2,* and *LcRS*, were identified and characterized in *L. chinense*. They shared high identities with their orthologous genes as well as shared common features related to plant riboflavin biosynthetic genes. LcRIBA, like other plant RIBAs, contains a DHBPS region in its N-terminal region and a GCHII region in its C-terminal part. It suggests that the enzymes specified by the plant gene are able to catalyze both the initial steps of the riboflavin biosynthetic pathway. LcLS1 was only 70% identical to the other isoform of the LS family in *L. chinense*, LcLS2. To our knowledge, isoforms in the same family of secondary metabolite biosynthetic genes normally share more than 80% similarity to each other. The divergence of LcLS1 and LcLS2 may be explained by the hypothesis that LS genes are synthesized in the cytosol with the N-terminal plastid-targeting sequences and then imported into plastids, where the N-terminal extension is removed to form the mature proteins [8,12,27]. As shown in Figure 2, the N-terminal extension of LcLS1 (amino acids 1–73) was only 30% identical to that of LcLS2 (aa 1–78), whereas the predicted mature part of LcLS1 (aa 74–233) shared high identity of 90% to that of LcLS2 (aa 79–237). LcRS also carried an N-terminal extension, which was believed to be a plastid-targeting sequence, that had low identity to the corresponding genes [8,28]. In addition, the N- and C-terminal domains of LcRS shared 23.7% identity and were found to have important implications in the disproportionation function [12].

QRT-PCR analysis showed that four riboflavin biosynthetic genes were constitutively expressed in all tissues examined of* L. chinense*, with the highest expression levels found in the leaves or red fruits. Starting from the beginning of the riboflavin biosynthetic pathway, *LcRIBA* was found at the highest levels in the leaves, which had the richest content of riboflavin. The expression level of *LcLS1* was strongest in the chloroplast organ (leaves), while that of *LcLS2* was highest in the chromoplast organ (red fruits). Similar to *LcLS2*,* LcRS* showed significantly higher expression in the red fruits compared to that in the other organs, while only a small amount of riboflavin was found in the red fruits. In addition, the four riboflavin biosynthetic genes showed significantly higher expression in the red fruits than in the green fruits; however, red fruits did not show higher accumulation of riboflavin content than green fruits. In our previous study, we also found that the transcription levels of riboflavin biosynthetic genes were not correlated with the accumulation of riboflavin in different tissues of *Momordica charantia* [13]. We hypothesize that there are several isoforms of riboflavin biosynthetic genes which have tissue-specific expression in plants. In addition, riboflavin in red fruits of *L. chinense* probably can be used as a substrate to synthesize its derivatives such as flavin mononucleotide and flavin adenine dinucleotide, causing the low content found in this organ. Further researches on the post-transcriptional processing or the accumulation of riboflavin derivatives are needed to explain why riboflavin accumulation is low in the red fruits of *L. chinense*.

## 3. Experimental Section

### 3.1. Plant Materials

*L. chinense*, cultivar Cheongmyeong, was grown in the experimental farm of Chungnam National University (Daejeon, Korea). After 6 months, the roots; stems; leaves; flowers; and fruits at two different stages, green fruits (immature) and red fruits (mature), were excised. All the samples were immediately frozen in liquid nitrogen and stored at −80 °C and/or freeze-dried for RNA isolation and/or HPLC analysis.

### 3.2. RNA Isolation and cDNA Synthesis

The samples were ground into a powder using a mortar and liquid nitrogen, and total RNA was isolated separately using a Plant Total RNA Mini Kit (Geneaid, New Taipei City, Taiwan) according to the manufacturer’s instructions. For first-strand cDNA synthesis, 1 μg of high-quality total RNA was used for reverse transcription (RT) by using a ReverTra Ace-R kit using oligo(dT)_20_ primer (Toyobo Co. Ltd., Osaka, Japan). A 20-fold dilution of 20 μL of the resulting cDNA was used as a template for quantitative real-time polymerase chain reaction (PCR).

### 3.3. Isolation of cDNAs Encoding Enzymes Involved in Riboflavin Biosynthetic Pathway

A total of 56,526 non-redundant cDNAs were obtained from *L. chinense* by using Illumina/Solexa HiSeq2000 DNA sequencing platforms in another study [29]. Of these, a partial-length cDNA encoding bifunctional GCHYII/DHBP, two full-length cDNAs encoding LS, and a full-length cDNA encoding RS were identified. These genes were then analyzed for homologies with known sequences and designated as LcRIBA, LcLS1, LcLS2, and LcRS (GenBank accession numbers: KF280343, KF280344, KF280345, and KF280346, respectively).

### 3.4. Sequence Analysis

The deduced amino acid sequences of LcRIBA, LcLS1, LcLS2, and LcRS were analyzed for homology by using the BLAST program at the NCBI GenBank database. Sequence alignments were performed using BioEdit Sequence Alignment Editor, version 5.0.9 (Department of Microbiology, North Carolina State University, Raleigh, NC, USA). The predicted molecular mass of proteins was calculated by using a program available online [30].

### 3.5. Quantitative Real-Time PCR

On the basis of the sequences of LcRIBA, LcLS1, LcLS2, and LcRS, we designed qRT-PCR primers using the Primer3 website [31] (Table 1). Real-time PCR products were tested for specificity of fragment sizes, melting curves, and sequences by PCR, real-time PCR, and cloning into a T-Blunt vector for sequencing, respectively. The expression of these genes was analyzed by the method of relative quantification by using the *L. chinense* actin housekeeping gene (KC810889) as a reference. For quantification of the standard, the PCR products that were amplified from cDNAs were purified, and the concentration of these products was measured to calculate the number of cDNA copies. The copy number of the Qrt-PCR standard was calculated as follows: concentration of PCR product (g/μL) × 10 − 9/[PCR product length in bp × 660] × 6.022 × 1023. Real-time PCR was conducted using a 20-μL reaction mix that contained 5 μL of template cDNA, 10 μL of 1× SYBR Green Real-time PCR Master Mix (Toyobo Co., Ltd., Osaka, Japan), 0.5 μL of each primer (10 μM), and diethylpyrocarbonate water. Thermal cycling conditions were as follows: 95 °C for 5 min, and 40 cycles of 95 °C for 15 s, 56 °C for 15 s, and 72 °C for 20 s. Each run contained a series of standards and a negative control (containing water instead of cDNA). PCR products were analyzed using Bio-Rad CFX Manager 2.0 software (Bio-Rad, Hercules, CA, USA). Three replications for each sample were used for the real-time analysis.

**Table 1:** Primers used for real-time PCR.

Primer	Sequence (5' to 3')	Amplicon (Base Pairs)
LcRIBA F	CTGGCTTAGACCCTGTTGGAGTAAT	169
LcRIBA R	GAAGCATGCTCTACCAACTGATCTC	
LcLS1 F	CAACTGTAATAAATCCTACGCAACG	157
LcLS1 R	CATGTTGTAAACCGGTTTAGATCCT	
LcLS2 F	CAATCCTTCACAGTTGCAACATTT	180
LcLS2 R	ACAGCTGATGTTTGAACTAAATCCC	
LcRS F	TTGAGCTTAAAACTGAAGGGGATTC	165
LcRS R	AAGCCACCAACATGAAGTTAAAACA	
LcActin F	ACCACTTGTTTGTGACAATGGAACT	198
LcActin R	TCAATTGGGTATTTCAAGGTCAAGA	

### 3.6. Riboflavin Extraction and Analysis

Riboflavin was extracted according to the method of Esteve* et al.* [32], with a slight modification. Briefly, vitamin B2 was released from the *L. chinense* samples (0.1 g) by adding 0.5 mL of 0.1 N hydrochloric acid, vortex mixing for 20 s, and placing in a water bath at 80 °C for 30 min. After cooling, the pH was adjusted to 4–4.5 using 2 M sodium acetate, and then, 0.1 mL of freshly prepared 10% (w/v) takadiastase solution in water was added. The mixtures were placed in a water bath at 50 °C for 3 h and then heated at 80 °C for 5 min. After cooling, 0.3 mL of water was added, and the extracts were filtered. Riboflavin was separated on a C18 column (250 × 4.6 mm, 5 μm; Symmetry RP18; Waters) by using an HPLC instrument (Shimadzu, Kyoto, Japan) equipped with a fluorometric detector (RF-10A; Shimadzu). The compound was detected on the basis of the λex/λem at 422/515 nm. Elution was performed using a binary gradient of 0.1% formic acid in water (mobile phase A) and 0.1% formic acid in acetonitrile (mobile phase B), according to the following program: 0 min, 95% A/5% B; 25 min, 65% A/35% B; 27 min, 5% A/95% B; 37 min, 5% A/95% B; 41 min, 95% A/5% B; and 51 min, 95% A/5% B. The flow rate was 1.0 mL/min, and the column temperature was 40 °C.

### 3.7. Statistical Analysis

The data for gene expression and riboflavin content were analyzed using the computer software Statistical Analysis System (SAS version 9.2, SAS Institute Inc., Cary, NC, USA). Treatment means were compared using Duncan’s multiple range test.

## 4. Conclusions

To date, riboflavin is mainly produced by biotechnological fermentation using bacteria or yeasts for over-production [33,34]; there are no reports on means of increasing riboflavin content in crop plants, the natural source of riboflavin for humans and animals. Thus, our finding may extend the understanding of the molecular mechanisms involved in the riboflavin biosynthesis, which will be helpful to identify key regulators controlling riboflavin accumulation in plants, leading to the successful metabolic engineering of vitamin B2 in crops in the near future.
